# Cranial vault metastasis revealing follicular thyroid carcinoma

**DOI:** 10.11604/pamj.2025.52.55.49268

**Published:** 2025-10-02

**Authors:** Sanae Habibi, Faycal El Guendouz

**Affiliations:** 1 Department of Endocrinology and Metabolism, Moulay Ismail Military Hospital, Meknes, Morocco; 2 Faculty of Medicine, Pharmacy, and Dentistry of Fez Sidi Mohammed Ben Abdellah University, Fez, Morocco

**Keywords:** Follicular thyroid carcinoma, cranial vault metastasis, multidisciplinary approach

## Image in medicine

We retrospectively report on a 52-year-old man who presented with intercurrent disease and signs of intracranial hypertension in our emergency department. He had a headache, vomiting, and photophobia. On clinical examination, a soft, non-tender parietal mass was noted, measuring 3 cm, without any inflammatory signs. Cerebral computerized tomography (CT) scan with contrast depicted an osteolytic bone lesion in the left parietal vault with mass effect on the adjacent brain parenchyma measuring 40.9×42×30 mm. Subsequent magnetic resonance imaging (MRI) confirmed the aggressive, expansile nature of the lesion. Ultrasound-guided biopsy of the lesion revealed cancer cells infiltrating into the bone, indicating bone metastasis of follicular thyroid carcinoma. Cervical US showed a solid hypoechoic thyroid nodule 73×40×45 mm (EU-TIRADS 4), excluding a plunging goiter. The patient was consulted for the ear, nose, and throat (ENT) department, and he was scheduled for total thyroidectomy with bilateral mediastinal recurrence lymph node dissection. Neurosurgical treatment entailed removal of the cranial metastasis and closure of the parietal defect using a local flap. Postoperative control was based on L-thyroxine suppressive therapy (dosed to suppress thyroid-stimulating hormone) with adjuvant high-dose radioiodine therapy (100 mCi). The clinical course was characterized by stabilization after the first administration of radioiodine therapy, with a clear improvement in functional performance up to resumption of gait. This case outlines a rare and sinister nature of cranial vault metastasis of follicular thyroid carcinoma and emphasizes the role of a multidisciplinary approach consisting of surgery, endocrinology, neurosurgery and nuclear medicine to achieve a satisfactory outcome.

**Figure 1:**
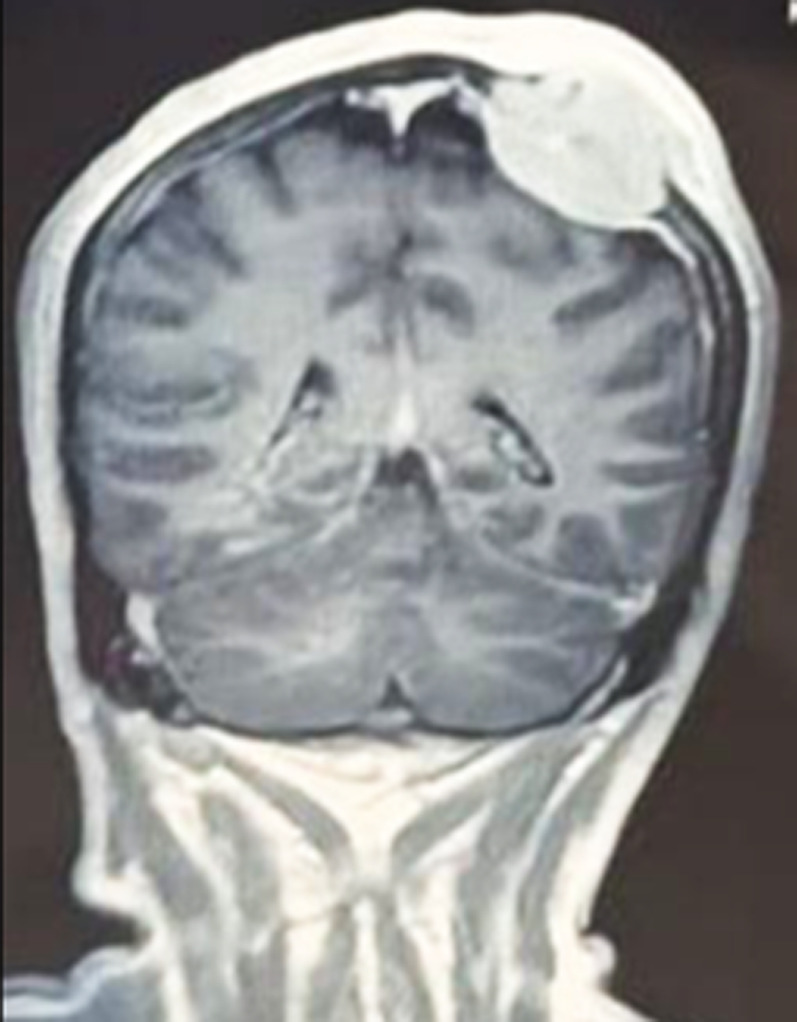
the brain magnetic resonance imaging in coronal section, T1-weighted sequence with gadolinium contrast, reveals a well-circumscribed extra-axial mass located at the right fronto-parietal convexity; the lesion demonstrates marked homogeneous enhancement and is in continuity with the dura mater; it exerts a mass effect on the adjacent cerebral parenchyma, causing mild displacement of the midline structures, with no evidence of parenchymal invasion visible on this section

